# Durable Vitality and Magical Forms

**DOI:** 10.3201/eid2805.AC2805

**Published:** 2022-05

**Authors:** Byron Breedlove

**Affiliations:** Centers for Disease Control and Prevention, Atlanta, Georgia, USA

**Keywords:** art science connection, emerging infectious diseases, art and medicine, about the cover, public health, viruses, complex viruses, helical viruses, filamentous viruses, isometric viruses, human immunodeficiency virus, SARS-associated coronavirus, MERS-CoV, hantaviruses, dengue virus, Ebola virus, Marburg virus, SARS-CoV-2, ribbon paintings, Lorser Feitelson, Magical Forms, Durable Vitality and Magical Forms, electron microscope, icosahedral

**Figure:**
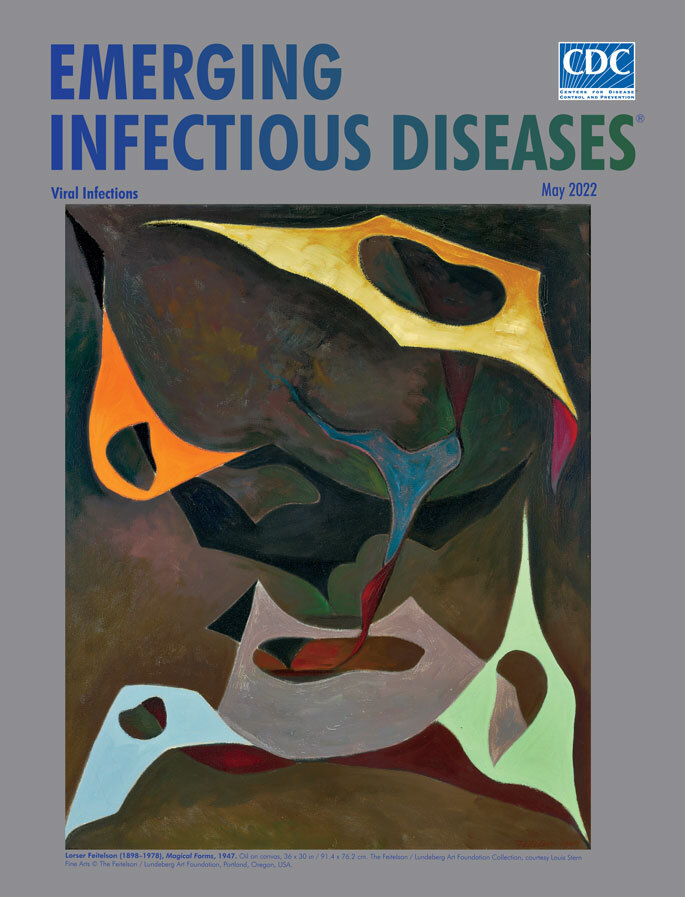
**Lorser Feitelson (1898–1978), *Magical Forms*, 1947.** Oil on canvas, 36 × 30 in/91.4 × 76.2 cm. The Feitelson /Lundeberg Art Foundation Collection, courtesy Louis Stern Fine Arts © The Feitelson/Lundeberg Art Foundation, Portland, Oregon, USA.

Lorser Feitelson grew up in New York City, and his interest in art was apparent when he was quite young. When young Feitelson was only six years old, his father started teaching him an analytical approach to drawing. His father’s extensive collection of books and periodicals provided the young artist the means to self-study classic and modern artwork. After attending the Armory Show of 1913—an event that included works by Cezanne, Van Gogh, Gauguin, Matisse, and Picasso, and is considered the beginning of Modernism in America―Feitelson decided to pursue painting as a career. At the age of 18, he rented a studio in Greenwich Village and from 1919−1927, made several trips abroad, living and studying in Paris.

In 1927, Feitelson moved to Los Angeles, where he lived and worked for most of his life. In addition to painting, he started what would be a 50-year career as an art instructor. Among his students was Helen Lundeberg, with whom he forged a working and a romantic relationship and married in 1956. The Smithsonian American Art Museum states, “Together, they adapted European surrealism into a new art movement known as subjective classicism. They rejected dreamlike free associations and instead placed objects together deliberately to evoke a particular idea.” The Feitelson/Lundeberg Art Foundation explains that their approach, also called Post-Surrealism, “did not rely on random, dream, personally symbolic, or arbitrary imagery. Instead, carefully planned objects or props were used to guide the viewer through the painting, gradually revealing a deeper and inter-connected meaning.” 

For a time, Feitelson may have been better known as the host of “Feitelson on Art,” his popular, unscripted, live television show that aired from 1956 through 1963, than for his artwork now found in many private collections, museums, and galleries. The Feitelson/Lundeberg Art Foundation notes that on his weekly TV show, “Over the years, Feitelson presented all eras, cultures and methods of making art from prehistoric through contemporary mid 1950s through early 1960s.” 

During 1940−1960, Feitelson experimented with abstract forms and his compositions shifted from organic imagery to geometric forms, culminating with his minimalistic “ribbon” paintings. This month’s cover image, *Magical Forms*, is one of several such paintings that share this title or variation of it. A series of tapered, twisting shapes, all with hollow centers, glide and drift across the canvas, emerging from and returning to the dark background. The brightly colored shapes along the edges appear to rise from the darkness, while the darker shapes seem to be receding. Their movements quietly evoke stingrays gliding under the ocean’s surface or bats pirouetting in twilight. The viewer is unsure what these magical forms represent, but the rhythm and balance of shapes and background are transfixing.

Feitelson’s notes on these paintings are telling: “There is nothing fortuitous or ‘automatic’ in the creation of these Magical Space Forms, fantastic though they are. Because I am concerned with durable vitality, rather than momentary frenzy, I find my work demands full participation of both my sensibilities and critical faculties.” 

During the 1890s, the same decade in which Feitelson was born, major breakthroughs occurred in virology. Dmitri Ivanovsky discovered that sap from diseased tobacco plants after being strained through filters that trapped bacteria could infect healthy plants, and Martinus Beijerinck, whose filtration experiments yielded the same results, named this pathogen a "virus.” In 1939, eight years after Ernst Ruska and Max Knoll built the first electron microscope, Ruska, Gustav Kausche, and Edgar Pfankuch used an electron microscope to record the first images of the tobacco mosaic virus with its rodlike shape. 

Before viruses were clearly understood and actually seen, they may have also seemed like magical forms. Viruses were determined to be submicroscopic parasites that cannot reproduce outside of a host and that have few components, essentially a single- or double-stranded nucleic acid and a protein coat in the form of a capsid. The structure and shape of viruses enable them to infect different types of cells and hosts, sometimes killing their hosts and sometimes coexisting without harming them. This high degree of specialization among viruses in a sense echoes Feitelson and Lundeberg’s notion of placing objects together deliberately to evoke a particular response. 

Given the abundance and diversity of viruses, it might have been expected that viruses would be found in a staggering array of shapes. After all, researcher Curtis Suttle notes, “If we compare the number of viruses in the oceans to the number of stars in the universe, there are about 10^23^ stars in the universe. In contrast, there are about 10 million-fold more viruses in the ocean than there are stars in the universe.” Infectious disease specialist David Pride wrote, “Biologists estimate that 380 trillion viruses are living on and inside your body right now—10 times the number of bacteria. Some can cause illness, but many simply coexist with you.”

Despite those nearly unfathomable numbers, most viruses are categorized as having one of three shapes: helical, icosahedral, or complex viruses. Helical viruses, or filamentous viruses, have rodlike, elongated shapes. Icosahedral viruses, or isometric viruses, possess 20 triangular sides or faces and 12 vertices, and 60 asymmetrical units. Complex viruses have multiple structural components that do not fit neatly into the other classifications. 

Since the 1980s, millions of people have been killed or sickened by a number of viruses, including human immunodeficiency viruses, coronaviruses, hantaviruses, hepatitis viruses, Ebola and Marburg viruses, dengue viruses, influenza viruses, and the measles virus. Because their evolution has yielded a wide diversity, viruses have maintained a durable vitality. Ensuring that public health infrastructure has a similar durable vitality for responding to emerging viral diseases and cyclic pandemics remains a high priority.
